# Draft genomes of *Klebsiella aerogenes*, *Klebsiella huaxiensis*, and *Klebsiella michiganensis* isolates from the urinary tract

**DOI:** 10.1128/mra.00492-24

**Published:** 2024-08-20

**Authors:** Alex Kula, Muna Arman, Helen Appleberry, Alan J. Wolfe, Catherine Putonti

**Affiliations:** 1 Department of Biology, Loyola University Chicago, Chicago, Illinois, USA; 2 Bioinformatics Program, Loyola University Chicago, Chicago, Illinois, USA; 3 Department of Microbiology and Immunology, Loyola University Chicago, Maywood, Illinois, USA; Wellesley College Department of Biological Sciences, Wellesley, Massachusetts, USA

**Keywords:** *Klebsiella*, *Klebsiella michiganensis*, *Klebsiella huaxiensis*, *Klebsiella aerogenes*, urinary tract infection, rUTI, urobiome, urinary microbiome

## Abstract

Several *Klebsiella* spp. can be the cause of urinary tract infections. Here we present the draft genome assemblies for four urinary isolates of three *Klebsiella* spp.: *Klebsiella aerogenes* UMB7541, *Klebsiella michiganensis* UMB11142 and UMB11423, and *Klebsiella huaxiensis* UMB11391 to further explore the genetic diversity of *Klebsiella* in the urinary tract.

## ANNOUNCEMENT

*Klebsiella michiganensis* and *Klebsiella huaxiensis* are members of the *Klebsiella oxytoca* complex (KOC). KOC species are common causes of urinary tract infections (UTIs), particularly among pregnant females [see review (1)]. *K. michiganensis* has been isolated from urine samples of both males and females with hospital-acquired UTIs (2). *K. huaxiensis* was first described from a urine sample of the cause of a patient’s UTI (3). *Klebsiella aerogenes* (previously *Enterobacter aerogenes*) also has been associated with UTIs (4). Prior research found that *K. aerogenes* and KOC species exhibit a higher incidence of antibiotic resistance than *E. coli* strains associated with UTIs (4, 5). Here we present the genomes of one strain of *K. aerogenes*, two strains of *K. michiganensis*, and one strain of *K. huaxiensis*, all isolated from catheterized urine samples. While the *K. aerogenes* strain was from a female diagnosed with recurrent UTI, the KOC strains were from three males, none of which presented with UTI symptoms at the time of collection.

Urine samples were collected as part of prior institutional review board (IRB)-approved studies [*Klebsiella aerogenes*: IRB 170077AW (University of California San Diego) (6, 7), *Klebsiella michiganensis* and *Klebsiella huaxiensis*: IRBs LU212677 and LU217801 (Loyola University Chicago)]. Each urine sample was processed using the expanded quantitative urine culture method (8). Microbial identification was determined using a matrix-assisted laser desorption ionization-time of flight mass spectrometer (Bruker Daltonics, Billerica, MA) as previously described (9). The isolates were stored at −80°C in the Loyola Urinary Education and Research Collaborative (LUEREC) collection. Samples were obtained from this collection and streaked on tryptone soy agar plates and incubated at 35°C in 5% CO_2_ for 24 hours. A single colony was selected and grown in liquid tryptone soy medium under the same culture conditions. The DNeasy Blood and Tissue Kit (Qiagen) was used to extract DNA following the manufacturer’s protocol for Gram-positive organisms. DNA was sent to SeqCoast Genomics (Portsmouth, NH). There libraries were constructed with the DNA Prep tagmentation kit (Illumnia) and sequenced with the Illumina NextSeq2000 platform (2 × 150 bp reads). Assembly was performed using the Bacterial and Viral Bioinformatics Resource Center (BV-BRC) web platform v.3.35.5 (10) with the “auto” parameter. There, the raw reads were trimmed using Trim Galore v.0.6.5 (https://github.com/FelixKrueger/TrimGalore) and assembled using Unicycler v.0.4.8 (11) followed by polishing with Pilon v.1.23 (12). Genome coverage was computed by BV-BRC. Genome annotations were produced using the National Center for Biotechnology Information (NCBI) Prokaryotic Genome Annotation Pipeline v.6.7 (13). Completeness and contamination were computed by CheckM v.1.2.2 (14) upon submission to the NCBI. Antibiotic resistance genes were identified using ResFinder v.4.5.0 (15, 16), specifying the *Klebsiella* species option. Default parameters were used for all software tools unless indicated otherwise.

Genome assembly statistics are provided in Table 1. All four strains are predicted to be resistant to cephalosporins. Table 1 lists the other antibiotic resistance genes identified. As this analysis shows, the three strains that are members of the KOC are predicted to be multi-drug resistant. Details about the patient’s antibiotic use prior to collection may provide insight into the predicted resistances observed.

**TABLE 1:** Genome statistics and strain details

Strain	UMB7541	UMB11142	UMB11423	UMB11391
Species	*K. aerogenes*	*K. michiganensis*	*K. michiganensis*	*K. huaxiensis*
SRA accession no.	SRR28710909	SRR28710906	SRR28710905	SRR28710900
Assembly accession no.	JBCGEN000000000	JBCGEJ000000000	JBCGEI000000000	JBCGEF000000000
No. of raw reads	2,252,548	2,518,876	2,472,466	3,746,804
Assembly length (bp)	5,283,186	6,024,287	5,951,917	6,220,856
G + C (%)	54.96	56.01	56.02	53.31
No. of contigs	44	63	117	92
Contigs N50 (bp)	378,967	407,338	174,411	284,828
Coverage (x)	60.78	60.29	58.91	83.91
Completeness (%)	95.91	99.48	99.47	98.3
Contamination (%)	4.42	0.94	0.42	2.81
Predicted antibiotic resistances^*a*^	Cephalosporins and carbapenem ompK36: 92.18%. Fosfomycin fosA: 90.05%.	Aminocyclitol aadA16: 99.64%. Aminoglycosides aac (3)-IId: 99.88%. aadA16: 99.64%. aac(6’)-lb-cr: 99.81%. aph(3’)-Ia: 100%. Beta-lactams bla_OXY-1-1_: 99.89%. bla_TEM-1B_: 100%. Cephalosporin: ompK36: 91.66%. Folate pathway antagonists dfrA14: 100%. sul2: 100%. Macrolide mph(A): 100%. Quinolones: aac(6′)-lb-cr: 99.81%. qnrB6: 100%. Rifamycin ARR-3: 100%.	Aminoglycoside aph(3′)-Ia: 98.89%. Beta-lactams bla_OXY-1-3_: 99.89%. bla_OXY-1-5_: 99.89%. Cephalosporin ompK36: 91.57%.	Beta-lactams bla_LAP-2_: 100%. bla_OXY-3-1_: 91.14%. Cephalosporin ompK36: 90.72%. Folate pathway antagonists dfrA14: 100%. sul2: 100%. Quinolone qnrS1: 100%. Tetracycline tet(A): 100%.

^*a*^ Percentage indicates the sequence identity to the ResFinder database sequence record.

## Data Availability

Table 1 lists the SRA accession numbers and assembly accession numbers for the four strains.
